# Capturing nystagmus during vertigo attacks using a smartphone: adherence, characteristics, pearls and pitfalls

**DOI:** 10.1007/s00415-023-11965-y

**Published:** 2023-08-31

**Authors:** Ali Melliti, Maurice van de Berg, Raymond van de Berg

**Affiliations:** Division of Balance Disorders, Department of Otorhinolaryngology and Head and Neck Surgery, Maastricht University Medical Centre, Maastricht, The Netherlands

**Keywords:** Nystagmocatcher, Ictal nystagmus, Nystagmus, Telemedicine, Telehealth, dizziness, Meniere disease, Benign paroxysmal positional vertigo

## Abstract

**Objective:**

To investigate adherence, characteristics, and first clinical experiences of capturing ictal nystagmus at home, which can be performed to complement the diagnostic process in patients with episodic vestibular symptoms.

**Methods:**

Patients were recruited at a tertiary referral center in case capturing ictal nystagmus could contribute to the diagnostic process (e.g., to detect or rule out BPPV). They were asked to capture ictal nystagmus with their own smartphone at home, using a smartphone-based adapter (Nystagmocatcher, Balansdiagnos, Stockholm, Sweden). All recordings were analyzed by the last author (RvdB), and the adherence, characteristics, and first clinical experiences were evaluated.

**Results:**

Seventy patients with vestibular symptoms were asked to participate in this study. Sixty-two (89%) agreed to participate. The median period of participation was 86 days. Fifty-one patients experienced attacks during the study period. Eventually, 51% of them provided eye movement recordings sufficient for analysis. Different types of nystagmus were observed: positional nystagmus related to BPPV, positional nystagmus not related to BPPV, functional eye movements, and the absence of nystagmus or functional eye movements. Capturing ictal nystagmus could contribute to the diagnostic process in several ways, including to detect or rule out BPPV, to detect or rule out vestibular origin of symptoms, to determine the affected side, telemedicine, to monitor attack frequency, and to detect malingering. Furthermore, strict guidance of patients was necessary, which could be time-consuming.

**Conclusion:**

Capturing ictal nystagmus can contribute to the diagnostic process in several ways, which motivates to rethink current clinical workflow in vestibular medicine. However, strict guidance is necessary and not all patients provide ictal recordings. In an outpatient setting, it would be advised to use ictal nystagmus recordings on indication, to complement the diagnostic process.

**Supplementary Information:**

The online version contains supplementary material available at 10.1007/s00415-023-11965-y.

## Introduction

Dizziness and vertigo are frequently encountered symptoms in daily clinical practice, which might result from vestibular disorders [1–3]. These vestibular disorders are classified into acute, episodic, and chronic vestibular disorders [4]. However, diagnosing episodic vestibular syndromes (e.g., benign paroxysmal positional vertigo, Menière’s disease) can be challenging. After all, since the attacks of vertigo and/or dizziness happen episodically, they are often not present when the patient visits the clinician: It can be difficult to ‘capture the moment.’ Unfortunately, reliable biomarkers to detect most of the vestibular disorders are still lacking [5]. Therefore, diagnosis of vestibular disorders still mainly relies on history taking, as reflected in the diagnostic criteria of vestibular disorders [6–19]. This imposes a diagnostic challenge, since patients often have difficulty to precisely describe their symptoms [20, 21].

Recently, different techniques of telemedicine were investigated to improve diagnostic accuracy of vestibular disorders. This included, e.g., standardized app-based diaries [22, 23], mobile hearing tests [24], and devices to capture ‘ictal nystagmus’ (nystagmus during attacks of vertigo and/or dizziness) [25–27]. Regarding this latter, it was demonstrated that ictal nystagmus can reliably be captured in up to 55% of patients with benign paroxysmal positional vertigo (BPPV), Menière’s disease, and vestibular migraine [25, 26]. In Menière’s disease and vestibular migraine, it was found that interictal nystagmus is horizontal in the majority of the cases (but can be vertical), and on average the slow-phase eye velocity of ictal nystagmus is higher in patients with Menière’s disease. Furthermore, reversal of the direction of ictal nystagmus can be present in a subset of patients with Menière’s disease [27].

In these studies, patients were only included after receiving a final vestibular diagnosis, and a specific video-oculography device was used to capture ictal nystagmus. This implies that determining the diagnostic value of capturing ictal nystagmus was (yet) beyond the scope of these studies. In addition, the use of specific video-oculography devices might compromise capturing ictal nystagmus in many clinics worldwide, due to the associated (currently relatively high) costs of the devices.

Objective of this study was therefore to capture ictal nystagmus in patients with episodic vestibular symptoms using a relatively inexpensive smartphone-based adapter (Nystagmocatcher, Balansdiagnos, Stockholm, Sweden) and to investigate:

1. Adherence: the number of patients (properly) recording their ictal nystagmus.
2. Characteristics: the characteristics related to the provided eye movement recordings, e.g., type of nystagmus.
3. First clinical experiences: pearls and pitfalls when implementing recordings of ictal nystagmus in daily clinical practice to complement the diagnostic process.

## Methods

### Study design

Patients were recruited at a tertiary referral center under the supervision of the last author (RvdB) between April 2020 and December 2022. This included patients in which capturing ictal nystagmus could contribute to the diagnostic process (e.g., to detect or rule out BPPV). One of the authors (MvdB) instructed the patients about the use of the Nystagmocatcher. They were asked to capture ictal nystagmus with their own smartphone at home, using a smartphone-based adapter (Nystagmocatcher). In case successful recordings were made and no more recordings were required from a diagnostic point of view, study participation ended. In case patients did not record ictal nystagmus in a period of 2 months, they decided together with two of the authors (RvdB, MvdB) whether the study was stopped or prolonged with, e.g., a couple of weeks or months. All recordings were analyzed by the last author (RvdB), and the adherence, characteristics, and first clinical experiences were evaluated.

### Patient characteristics

Patients were prospectively convenience recruited at the Department of Otorhinolaryngology and Head and Neck surgery of Maastricht University Medical Center, The Netherlands. Inclusion criteria comprised: (1) visiting the outpatient clinic because of a history of attacks of vertigo and/or dizziness in the last 2 months, and (2) the performed standard investigations were not adequate to complete the diagnostic process. These investigations included structured history taking, neuro-otological examination, positional testing, audiometry (pure-tone and speech), vestibular laboratory testing (video head impulse test, bithermal caloric test and torsion swing test), and imaging on indication (e.g., an MRI to rule out a vestibular schwannoma); (3) having access to a smartphone. Patients were excluded in case they indicated, at the time of recruitment, that recording ictal nystagmus would not be possible for them (e.g., too little skills to operate the video software of the smartphone).

### Device: The Nystagmocatcher

**Fig. 1:**
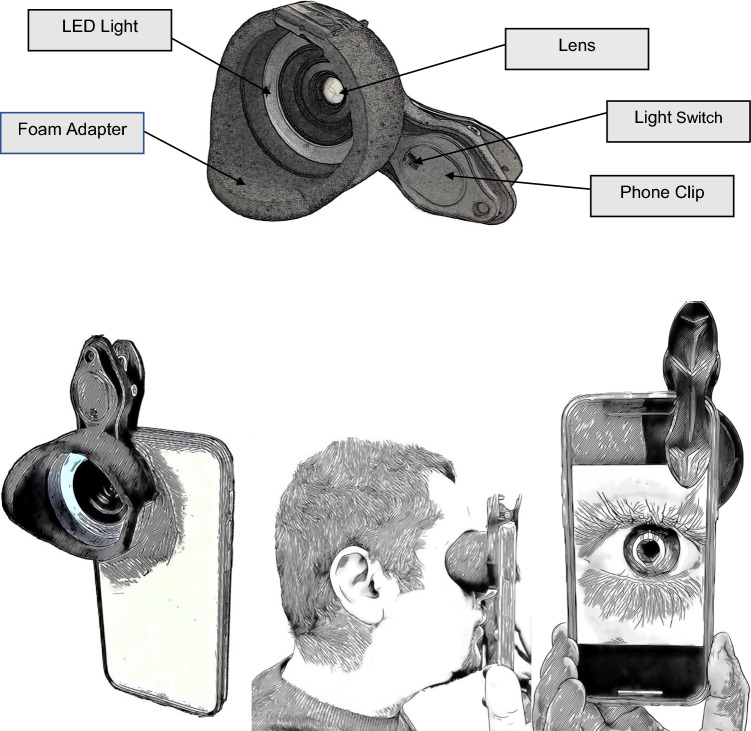
The Nystagmocatcher. The Nystagmocatcher can be coupled to any commercially available smartphone. It is placed in front of the eye and a video recording can be made with the smartphone

The Nystagmocatcher is a smartphone-based adapter, which enables a close view of the eye using a commercially available smartphone (Fig. 1). It comprises a 3D-printed plastic frame which contains a LED light to illuminate the eye and a + 15 Diopter lens which is placed in front of the lens of the smartphone. In addition, a foam ring is attached to prevent (as much as possible) light from the outside entering the space between the patient’s eye and the lens of the Nystagmocatcher. The Nystagmocatcher is placed in front of one of the eyes, while a video is made by the hardware and software of the smartphone.

### Instructions and guidance during the study period

Within 1 week after recruitment, all patients were instructed and guided by one of the authors (MvdB). This included several contacts.

The first contact comprised verbal and written instructions about the use of the Nystagmocatcher and additional questions about baseline characteristics. Patients were asked to record eye movements during discrete attacks of vertigo and/or dizziness (i.e., attacks with a clear beginning and end) at home. The instructions included (1) recording eye movements in sitting upright position, while looking forward, to the right and to the left, each for 20 s; (2) recording eye movements during the Dix-Hallpike maneuver to the left, and to keep recording until 20 s after this maneuver; (3) recording eye movements during the Dix-Hallpike maneuver to the right and to keep recording 20 s after this maneuver. Additionally, patients were asked to avoid blinking during the recordings and to use an eye patch to occlude the non-recorded eye (Opticlude, 3 M, Maplewood, USA). Not all patients were instructed to record in all positions: The protocol was tailored to the patient’s situation. For example, patients in which BPPV was ruled out before did not have to record the Dix-Hallpike maneuvers. In some specific cases, patients were asked to repeat the recordings at 45 min to 2 h after the onset of the attack. This involved cases in which reversal of the direction of the ictal nystagmus could help in identifying the affected/active side (e.g., patients with disabling attacks due to bilaterally affected vestibular organs, in which the active side was not clear). Finally, it should be noted that patients were specifically instructed to only submit videos to the authors in case the recordings were made during an attack of vertigo and/or dizziness: The vertigo and/or dizziness needed to be experienced during the recordings.

The second contact was scheduled two days after the first contact. The aim of this study was to verify whether patients still wanted to participate after receiving the extensive instructions. If not, participation stopped.

After the first and second contacts, patients were always contacted after they submitted their recordings. The next steps were discussed, for example stop participation if the quality of the recordings was sufficient, and make new recordings if recordings were of insufficient quality.

In case patients did not record any videos, patients were contacted each month. During these follow-up contacts, patients were asked whether they wanted to proceed with the study and how many attacks occurred in the last month. Besides, reasons for not recording attacks (if any occurred) were inquired.

Additionally, patients could always contact one of the authors (MvdB) for questions or support related to use of the Nystagmocatcher or study participation.

### Analysis of ictal nystagmus video recordings

All eye movement recordings were analyzed by the same author (RvdB) on a 4 K 32-inch monitor (LG 32UN88A, Seoul, South Korea). Eye movement recordings were classified as ‘sufficient for analysis’ in case the patient’s eyes were visible enough to be able to determine the presence of nystagmus and whether all steps of the protocol were sufficiently followed (e.g., in case of suspected BPPV, the Dix-Hallpike maneuvers needed to be performed). Eye movement recordings classified as ‘insufficient for analysis’ were not used for evaluation of ictal nystagmus. The first provided eye movement recordings, sufficient for analysis, were used. This was decided in order to have an equal representation of all patients, regardless of the number of recordings they submitted. Main findings of the eye movement recordings were obtained and classified into spontaneous horizontal nystagmus, spontaneous vertical nystagmus, positional nystagmus related to BPPV (positive Dix-Hallpike maneuver), positional nystagmus not related to BPPV, functional eye movements, and no nystagmus or functional eye movements. Main findings were defined as the ‘most clinically relevant findings’ of the provided recordings. For example, a spontaneous horizontal nystagmus in upright position which increased in amplitude during the Dix-Hallpike maneuvers was classified as ‘spontaneous horizontal nystagmus,’ and not classified as ‘positional nystagmus not related to BPPV.’ This latter was only used in the absence of spontaneous nystagmus in upright position. Eye velocities of ictal nystagmus were explicitly not calculated, to keep the study as simple as possible without too many technical demands (considering future implementation in clinics worldwide).

### Data analysis

Microsoft Excel (Microsoft, Redmond, USA) and SPSS (IBM, Armonk, New York) were used for data analysis and RStudio (Posit PBC, Boston, Massachusetts) to generate the bar charts. Descriptive statistics were applied. In case patients agreed to participate at time of recruitment, but declined participation directly after the instructions, this was considered as ‘no participation.’ Patients were included in the analysis in case they had the opportunity to record their eye movements during a period of at least 2 months. As stated above, follow-up could be shorter if sufficient eye movements recordings were provided within 2 months.

Non-adherence was categorized into non-fulfillment and non-compliance [28]. Both categories comprised patients who agreed to participate after the first instructions. In case of non-fulfillment, this involved patients who did not record any eye movements, despite having attacks. In case of non-compliance, this involved patients who did not record any eye movements *sufficient for ictal nystagmus analysis*, despite having attacks. It was decided to not analyze the percentage of attacks in which ictal nystagmus was recorded, since during this study it was found that patients had difficulty to reliably indicate the amount of attacks during a specific time period, which was congruent with previous literature [22].

Reasons for not participating and not recording eye movements were categorized in consensus by two of the authors (MvdB, RvdB). First clinical experiences were discussed between all authors in multiple meetings. These experiences were categorized and described in consensus.

## Results

### Patient characteristics

Seventy patients with vestibular symptoms were asked to participate in this study. Sixty-two of them (89%) agreed to participate after the instructions. This comprised 24 males and 38 females, with a mean age of 48 years (minimum 15 years, maximum 73 years). The eight patients declining participation involved three males and five females, with a mean age of 64 years (minimum 36 years, maximum 82 years). The median period of participation was 86 days (interquartile range 52–129 days, minimum 8 days, maximum 246 days).

Thirty-one patients eventually provided eye movement recordings (see below). Etiologies of these patients comprised Menière’s disease (*n* = 7), episodic vertigo not otherwise specified (*n* = 3) [29], BPPV (*n* = 5), functional vestibular disorder (*n* = 5), hydropic ear disease, not related to Menière’s disease (*n* = 4) [30], unclear diagnosis (*n* = 3), vestibular migraine (*n* = 2), auto-immune inner ear disease (*n* = 1), and panic disorder (*n* = 1).

### Adherence

#### Reasons for declining participation

**Fig. 2:**
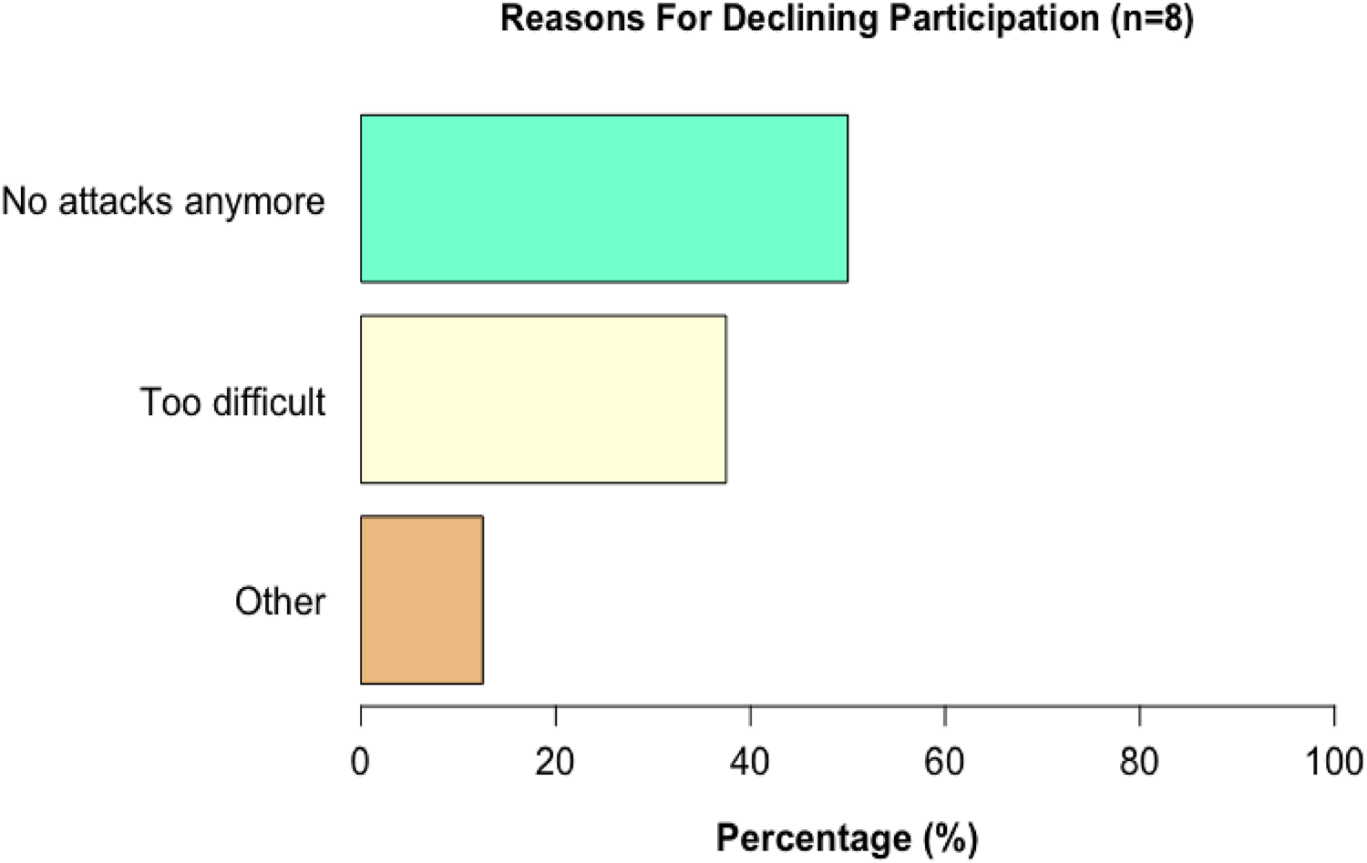
Reasons for declining participating in this study

Figure 2 presents the reasons for declining participation in this study (*n* = 8). These reasons included: vestibular symptoms disappeared in the period between recruitment and instructions (‘no attacks anymore,’ 50%); recording ictal nystagmus according to the instructions was considered too difficult (‘too difficult,’ 38%); and ‘other’ (13%).

#### Reasons for not providing eye movement recordings

**Fig. 3:**
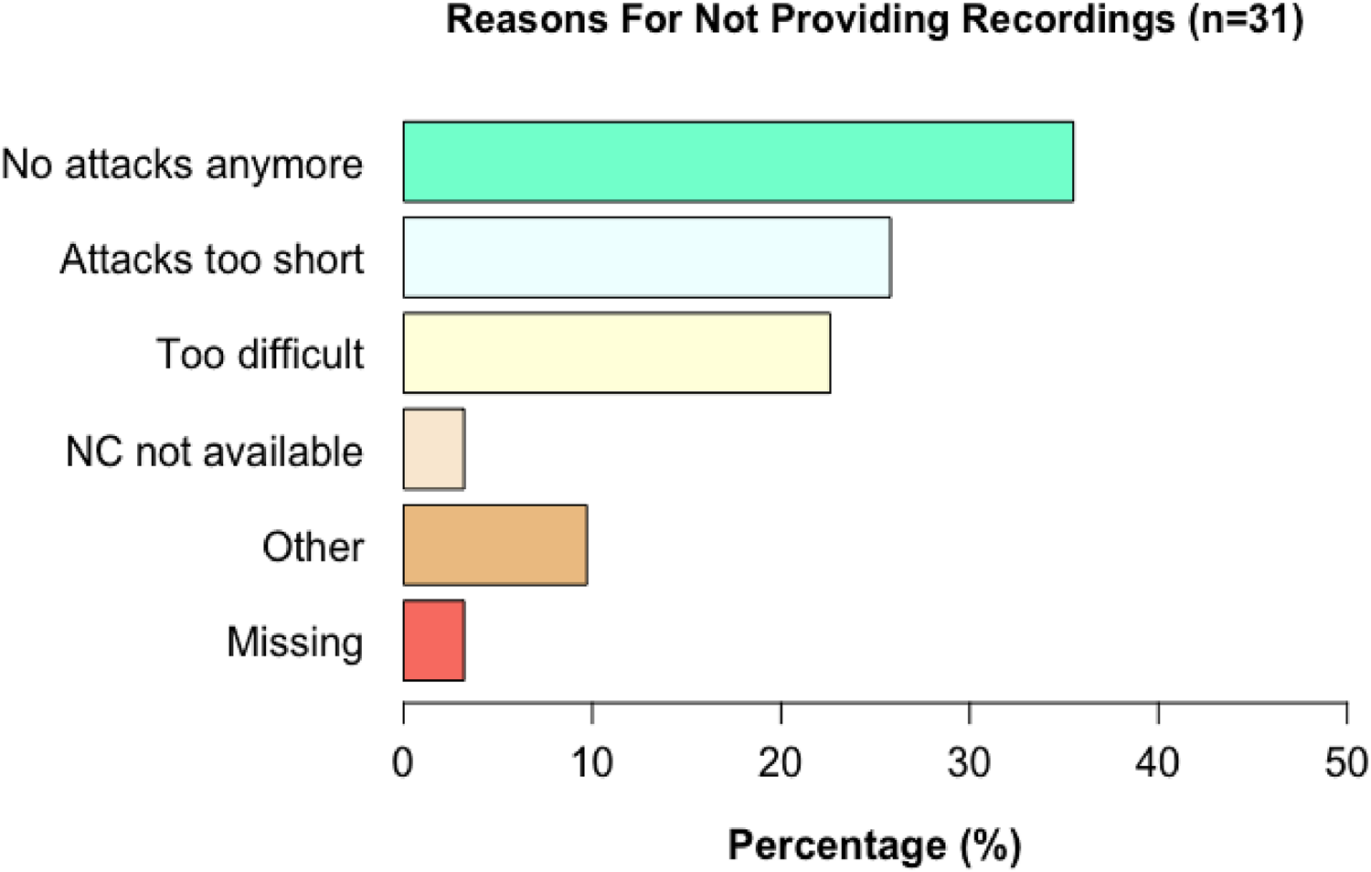
Reasons for not providing eye movement recordings of attacks of vertigo and/or dizziness. *NC*  Nystagmocatcher

Thirty-one patients (50%) provided eye movement recordings. Reasons for not providing eye movement recordings of attacks of vertigo and/or dizziness are demonstrated in Fig. 3. Main reasons included no attacks anymore during the study period (36%); too difficult (23%); and attacks were too short to be able to record them properly (‘attacks too short,’ 26%). One patient did not have the Nystagmocatcher available during an attack (3%). The reasons listed as ‘other’ were feeling ashamed (3%), family circumstances (3%), and experiencing only mild vestibular symptoms (3%).

#### Provided eye movement recordings: non-fulfillment and non-compliance

Sixty-one percent of patients who experienced attacks of vertigo and/or dizziness during the study period (31 out of 51) provided eye movement recordings, resulting in a non-fulfillment of 39%. Eventually, 51% of patients with attacks of vertigo and/or dizziness (26 out of 51) provided eye movement recordings sufficient for analysis, resulting in a non-compliance of 49%. In the non-compliant cases, instructions were not followed (e.g., no eye movements recorded despite having attacks, only recordings in sitting upright position without positional maneuvers, eye movement recordings when having no attack). Initially, eight patients provided recordings which were insufficient for analysis. However, after new instructions, three out of these eight patients eventually sent new recordings sufficient for analysis. An unclear diagnosis remained in three out of five patients who provided only insufficient recordings.

### Characteristics related to provided eye movement recordings

Regarding the 31 patients who provided eye movement recordings, the reasons to indicate eye movement recordings were complementing the differential diagnosis process (*n* = 26), determining the affected side (*n* = 4), and follow-up in BPPV (*n* = 1).

The median time to provide the first eye movement recording was 17 days (interquartile range 9–48 days, minimum 1 day, maximum 190 days). The median time to provide the first eye movement recording sufficient for analysis and diagnosis was 20 days (interquartile range 9–67 days, minimum 1 day, maximum 190 days). Table 1 illustrates the main findings of the first provided eye movement recordings of all patients, which were sufficient for analysis.

**Table 1:** Main findings of first provided eye movement recordings, sufficient for analysis

Eye movement findings	Number (*n*)	Percentage (%)
Spontaneous horizontal nystagmus	15	58
Spontaneous vertical nystagmus	1	4
Positional nystagmus related to BPPV (Positive Dix-Hallpike maneuver)	4	15
Positional nystagmus not related to BPPV	2	8
Functional eye movements	1	4
No nystagmus or functional eye movements	3	12

Additionally, five patients provided two separate recordings at different time intervals (different attacks). In two patients with hydropic ear disease, the first recordings showed spontaneous horizontal nystagmus and the second recordings showed spontaneous vertical (downbeat) nystagmus. In the remaining three patients, findings were consistent in both recordings: a spontaneous horizontal nystagmus (*n* = 2) and a positional nystagmus related to BPPV (*n* = 1).

### First clinical experiences: pearls and pitfalls

Multiple pearls and pitfalls related to recording ictal nystagmus were identified during this study. Table 2 presents the diagnostic pearls, which includes indications to use ictal nystagmus recordings and additional findings that could facilitate the diagnostic process (e.g., prospectively monitoring attack frequency).

**Table 2:** Diagnostic Pearls related to recording ictal nystagmus

Pearl	Cases (examples)	
	History	Main findings ictal nystagmus recordings
Detect or rule out BPPV	Woman, 31 years, typical history of recurrent BPPV, never observed during testing at clinic(s)	Upper poli of the eye rotates toward the left ear during Dix-Hallpike to the left (case 1, appendix), indicating BPPV posterior canal left
	Man, 55 years, recurrent short vertigo attacks, trigger unclear (spontaneous or head movements)	Spontaneous horizontal/torsional nystagmus to the right, in sitting upright position (case 2, appendix), ruling out BPPV
Detect or rule out vestibular origin	Woman, 17 years, recurrent vertigo attacks after otologic surgery. Functional origin is suspected, but vestibular origin is not ruled out	No nystagmus observed during attacks (case 3, appendix). Functional origin becomes more likely
Determine affected side	Man, 68 years, recurrent vertigo attacks with bilateral otosclerosis and secondary hydrops	Spontaneous horizontal/torsional nystagmus to the right, in sitting upright position (case 4, appendix, ‘irritative phase’). One day after another attack: spontaneous horizontal nystagmus to the left (observed in clinic, ‘paretic phase’). Findings indicate the right vestibular organ as the affected/active side
Telemedicine	Woman, 50 years, diagnosed with bilateral BPPV. Treated for both sides with Epleys. Travel distance more than 2.5 h from clinic. Follow-up of treatment	Upper poli of the eye rotates toward the right ear during Dix-Hallpike to the right (case 5, appendix). Dix-Hallpike to the left negative, indicating BPPV posterior canal right
Prospectively monitor attack frequency	Woman, 24 years, reports at least 20 vertigo attacks in the 2 months before start of study	No recordings: no attacks anymore during study period
Detect ‘malingering’	Woman, 46 years, reports up to 3 disabling vertigo attacks per week A vestibular origin is initially suspected	No recordings, avoiding contact. Eventually reports it to be ‘too difficult,’ but also refrains from contact to discuss treatment (at least until time of writing). Malingering is suspected

Table 3 illustrates the logistic pearls identified during this study. Logistic pearls are mainly intended to improve adherence, which is crucial to obtain proper recordings.

**Table 3:** Logistic pearls related to recording ictal nystagmus

Pearl	What to do?	Background
Protocol tailored to situation	Instruct to record the minimum necessary, e.g.: - *Interested in BPPV*: Only record positional testing - *Interested in spontaneous attacks*: Only record spontaneous nystagmus - *Differentiate BPPV from other attacks*: Record spontaneous nystagmus and positional testing	Recording ictal nystagmus was often reported as a burden. Fewer recordings decrease the burden and might increase the adherence
Proper patient selection	Only select patients in which recording ictal nystagmus benefits the medical process	Recording ictal nystagmus is also time consuming for the clinician: proper guidance is necessary. If chance is low that proper recordings will be made, the benefits (proper recordings and diagnosis) might not outweigh the costs (time for guidance)
	Only select patients who are motivated to record ictal nystagmus. Check their motivation	
	Only select patients who know how to operate a smartphone (or have access to help)	
	Only select patients who are still experiencing attacks of vertigo and/or dizziness	If patients are free of attacks for a longer period of time (e.g., more than 2 months), attacks might stay away for a longer period of time [31]. It could be difficult to obtain recordings
Proper guidance	Explain why recording ictal nystagmus is necessary	Patients’ understanding of the concept increases adherence [32, 33]
	Explain the definition of an attack: it should have a clear beginning and end	Only attacks should be recorded
	Explain that the vertigo and/or dizziness should be experienced during the recordings	Avoid that patients provide recordings which are not really made during an attack (e.g., BPPV might have disappeared already when getting ready for the recordings)
	Instruct patients to verbally describe their head position during the Dix-Hallpike maneuvers [26]	In some recordings it can be difficult to determine which Dix-Hallpike maneuver was recorded
	Provide verbal and written instructions	Verbal instructions enable patients to ask questions. Written instructions provide step-by-step guidance during the recordings. This improves adherence [32]
	Ask for help of a partner, friend, or physical therapist, if necessary	Patients might be too sick, too inexperienced with a smartphone, or unable to perform the positional maneuvers by themselves, to properly record videos during an attack. Help from others facilitates proper recordings

Table 4 demonstrates the pitfalls identified during this study. Additionally, mitigation strategies used to avoid re-occurrence of these pitfalls, are mentioned.

**Table 4:** Pitfalls related to recording ictal nystagmus

Pitfall	Background	Mitigation strategy
Patients do not record any videos	Many factors can contribute to not recording any videos	Schedule regular follow-ups and investigate (and try to mitigate) reasons for not recording any videos [32] *Example: consider prolonging study period in case of attack-free interval*
Patients record videos when not having attacks	Patients did not understand instructions properly	Always check whether: 1) Recordings were made during an attack 2) Vertigo was experienced during recording If not the case: instruct again
Too little devices (Nystagmocatchers) to facilitate recordings	Patients can have a device for many months. It is not available for other patients during this period	Have enough devices available
Attacks too short to be able to record	Some disorders (e.g., Vestibular Paroxysmia) are characterized by attacks which already stop before recording is started	No perfect mitigation strategy found yet. These patient groups might be less qualified for ictal nystagmus recordings using a smartphone
Patients record spontaneous nystagmus	Patients can have spontaneous nystagmus, in addition to ictal nystagmus. Spontaneous nystagmus can be confused with ictal nystagmus when analyzing recordings	Always check the presence of spontaneous nystagmus, before analyzing ictal nystagmus recordings. If present: take it into account when analyzing ictal recordings
Capturing direction-reversal of ictal nystagmus fails	(1) Patients can consider it a burden to record multiple times during an attack (2) The time until direction-reversal of nystagmus differs between patients, and it is not always present [27]	Only try to record direction-reversal of ictal nystagmus unless it benefits the medical process (e.g., to determine the side for therapy in bilateral Menière’s Disease) If indicated: record multiple times during an attack (e.g., each 15 min) [27]

## Discussion

The objective of this study was to investigate adherence, characteristics, and first clinical experiences of capturing ictal nystagmus at home, which can be performed to complement the diagnostic process in patients with episodic vestibular symptoms. It was found that 51% of the patients with attacks of vertigo/and or dizziness, provided eye movement recordings sufficient for analysis. In these patients, different types of nystagmus were found, depending on the disorder. Capturing ictal nystagmus could contribute to the diagnostic process in several ways, including to detect or rule out BPPV, to detect or rule out vestibular origin of symptoms, to determine the affected side, telemedicine, to monitor attack frequency, and to detect malingering. Furthermore, strict guidance of patients can improve results, but might be time-consuming. In an outpatient setting, it would therefore be advised to use ictal nystagmus recordings on indication, to complement the diagnostic process.

Adherence in this study involved a compliance of 51%, which is congruent with previous literature [25]. This implies that a significant number of patients does not provide any eye movement recordings. The main reason for not providing eye movement recordings was the non-recurrence of attacks of vertigo and/or dizziness (36%). This is lower than in a previous study (73%), which might be related to the clinical setting (ENT versus neurology outpatient clinic) [25]. Nevertheless, other reasons like having only very short attacks or finding it too difficult to record eye movements during attacks should not be disregarded. Most likely, some disorders (e.g., vestibular paroxysmia) and patient groups (e.g., people not familiar with operating a smartphone) might remain less qualified for ictal nystagmus recordings using a smartphone. Regarding the latter, patients were therefore not included in this study if it was evident that they were not able to operate a smartphone properly.

The median time to provide the first eye movement recording was 17 days, which implies that the majority of patients will provide a recording within 1 month. However, some patients can have attack-free intervals, and need to loan the recording device for a longer period of time. This suggests that in an outpatient setting, multiple devices need to be obtained, to be able deploy them in different patients during the same time period. Therefore, these devices should preferably be inexpensive.

Different types of ictal nystagmus were found in the provided eye movement recordings, varying from spontaneous horizontal and vertical nystagmus to positional nystagmus related to BPPV, positional nystagmus not related to BPPV, functional eye movements, and the absence of nystagmus or functional eye movements. These findings were used to complement the diagnostic process (see below). Strikingly, two patients with hydropic ear disease showed different alignments of their ictal nystagmus (spontaneous horizontal and spontaneous vertical (downbeat) nystagmus) in two separate recordings at different time intervals (different attacks). The presence of ictal spontaneous vertical nystagmus (mainly downbeat) was previously described to be around 7% in patients with Menière’s disease [27]. In this study, this type of nystagmus was found in three out of four patients with hydropic ear disease and therefore in 3 out of 11 patients with hydrops-related disorders (27%). This might indicate that the prevalence of ictal spontaneous vertical nystagmus could be higher than previously reported. It also implies that an ictal spontaneous vertical nystagmus does not directly indicate a central vestibular disorder in all patients. Furthermore, it was illustrated that in some patients more than one successful recording might be necessary, to obtain a full clinical picture. After all, different types of nystagmus can be present within the same patient.

Main objective of capturing ictal nystagmus is to complement the diagnostic process. It is able to ‘capture the moment’ in patients with episodic vestibular symptoms, which is not always possible in a regular outpatient setting. As stated above, this study identified several ways on how capturing ictal nystagmus can contribute to the diagnostic process (see Table 2).

Firstly, it can be used *to detect or rule out BPPV.* Only in these cases, ictal nystagmus recordings can directly provide a diagnosis, since the typical nystagmus can be observed (e.g., crescendo-decrescendo nystagmus with the upper poli of the eye rotating toward the lower ear and vertically toward the forehead during the Dix-Hallpike maneuver, in posterior canal BPPV [9]). Capturing ictal nystagmus at home can be indicated in BPPV in the following cases:

- History taking suggests BPPV or at least the need to rule out BPPV (e.g., vestibular migraine patient with positional vertigo [34]), but diagnostic maneuvers at the outpatient clinic do not show any objective evidence of BPPV. Since the Dix-Hallpike and lateral roll can show false-negative results, it is advised to repeat the maneuvers at a separate time to confirm the diagnosis [35]. When symptoms reoccur, ictal nystagmus can now be recorded at any location using a smartphone. This increases the chance of detection, especially in short-lived episodes of BPPV (‘capture the moment’).
- Recurrent episodes of BPPV and the patient living far away from the outpatient clinic. By capturing ictal nystagmus at home, a *telemedicine approach* can be used to verify recurrence of BPPV and to determine the affected canal. The proper treatment can be proposed remotely, without the need of the patient travelling to the outpatient clinic [36].

Secondly, capturing ictal nystagmus can be used to *detect or rule out a vestibular origin* of symptoms.

This is mainly indicated when history taking, physical examination and (on indication) ancillary testing were unsuccessful in reliably classifying the symptoms in one of the vestibular disorders or their non-vestibular differential diagnoses. In these cases, capturing ictal nystagmus could show objective evidence of the presence of nystagmus, indicating a (central or peripheral) vestibular disorder, depending on the type of nystagmus. This would make other non-vestibular disorders, like cardiac arrhythmia, hypoglycemia, or panic attack [6], less probable. It should be noted that the absence of nystagmus does not rule out a vestibular disorder, but it might make it less likely. However, when no nystagmus is observed during recordings, it is advised to double-check whether vertigo was really experienced during recording. After all, some patients can record eye movements when having no attack, or only very little symptoms (Table 4). In these situations, nystagmus may not be present, leading to false-negative recordings. In case capturing ictal nystagmus is used to detect or rule out a vestibular origin of symptoms, the recordings most likely not directly provide the diagnosis, in contrast to recordings in BPPV patients. Nevertheless, in the future it might contribute to classifying vestibular disorders based on nystagmus characteristics. For example, the average slow-phase eye velocity of ictal nystagmus is higher in patients with Menière’s disease than in patients with vestibular migraine. It was therefore previously demonstrated that combing ictal nystagmus velocity and caloric canal paresis correctly separates a diagnosis of Menière’s disease from vestibular migraine with a high sensitivity and specificity (96% and 85%, respectively) [27].

Thirdly, capturing ictal nystagmus can be used to *determine the affected side*. In BPPV patients, the affected side (and canal) can reliably be determined based on nystagmus characteristics during diagnostic maneuvers [35]. However, in other vestibular disorders, especially (bilateral) Menière’s disease, this can be much more challenging. After all, during an attack, an ‘irritative’ (ipsiversive), ‘paretic’ (contraversive) and ‘recovery’ (ipsiversive) nystagmus can be found, depending on the patient and duration of attack. Approximately 59% of Menière’s disease patients show direction-reversal nystagmus, which can be ipsiversive to contraversive, but also contraversive to ipsiversive. Of these 59%, more than half demonstrate direction-reversal in the first 12 h (mean irritative-paretic direction reversal latency of 22 min) and 41% over different days. Additionally, in patients without direction-reversal findings, approximately 2/3rd demonstrates ipsiversive nystagmus, while 1/3rd demonstrates contraversive nystagmus [27]. In other words, it can be difficult to determine the affected side, since it is not always clear whether the recordings were made in the irritative, paretic, or recovery phase. A better insight into the evolution of the phases during an attack could be obtained by continuously recording eye movements using an ambulatory device [37]. If it is preferred to use smartphone recordings, ‘semi-continuous’ recordings can be obtained, by, e.g., recording eye movements each 15 min from the onset of the attack, until it ends [27]. However, since this might be considered a burden, it could lead to reactivity and undersampling of attacks of vertigo and/or dizziness [38].

Fourthly, capturing ictal nystagmus can *prospectively monitor the attack frequency* and provides, compared to history taking in clinic, a more reliable understanding of the number of attacks of vertigo and/or dizziness. After all, capturing ictal nystagmus is an example of event sampling: A clearly defined event (an attack of vertigo and/or dizziness) should be recorded. Event sampling involves less retrospectivity (and therefore, recall bias) than history taking, because the latter is generally performed at a later stage [22]. Since recall bias includes the tendency to report peak symptom scores [39], it might be hypothesized that history taking could lead to overreporting of attacks [22]. It should be noted that event sampling is more prone to under-sampling due to the additional workload which is involved in recording eye movements [38]. This can (partially) be mitigated by applying recording protocols tailored to the situation (Table 3): Fewer recordings might increase the adherence. Additionally, prospectively monitoring the attack frequency could also *detect malingering*, since patients are confronted with the request to provide objective findings of their symptoms.

These first clinical experiences motivate to rethink the current clinical workflow in vestibular medicine. After all, capturing ictal nystagmus can be complementary in the diagnostic process but is not (yet) routinely used in many clinics worldwide. In BPPV, it can establish a diagnosis, but in other disorders it provides the opportunity to think beyond simply ‘getting the diagnosis,’ e.g., confirming or ruling out a vestibular origin in case of doubt, ruling out a specific vestibular disorder in case of doubt, determining the affected side in case of bilateral vestibular involvement, and telemedicine. This could present a major step forward in vestibular medicine, since it provides information complementary to history taking, physical examination, and ancillary testing. Capturing ictal nystagmus is a diagnostic tool which is able to reliably ‘capture the moment’ and show objective evidence of (central or peripheral) vestibular involvement in short-lived episodes of vertigo and/or dizziness. It might even be obtained at home, before the first visit to clinic. Furthermore, the objective presence of ictal nystagmus is not (yet) part of many diagnostic criteria of episodic vestibular disorders, such as Menière’s disease, vestibular migraine, or vestibular paroxysmia [13, 34, 40]. Future research could focus on the prevalence of ictal nystagmus in these disorders. If prevalence would be high, adding the presence of ictal nystagmus in the diagnostic criteria could be considered. This might increase specificity, but also sensitivity by having an objective finding of nystagmus as an additional criterion which can be met (e.g., criterion A, OR ictal nystagmus).

Finally, guidance of the patient is very important. It is a necessary and time-consuming activity, which is required to ensure that patients properly record their eye movements in all positions, when having severe symptoms at the same time. Precise instructions and good understanding of the patient are crucial [36]. It would therefore be advised to provide verbal and written instructions and to remain in close contact with the patient, especially after they provided videos insufficient for analysis. By this, adherence can be improved [32]. However, the time needed for guidance (instructions and follow-up) should be emphasized: In this study, several contacts were necessary. Guidance took more than 45 min for each patient. It would therefore be advised to only use ictal nystagmus recordings in case objective findings of nystagmus are considered to complement the diagnostic process. In the future, it could be hypothesized to use smartphone-based applications which could instantaneously provide the proper instructions for capturing ictal nystagmus during an attack of vertigo and/or dizziness. Possibly, these applications might also be able to analyze the eye movements using, e.g., a deep learning system [41].

### Limitations

Three limitations were identified in this study. First, the Nystagmocatcher uses the smartphone camera. Conventional smartphone cameras do not use infrared. Therefore, the eye is not measured in complete darkness, and some suppression of the vestibulo-ocular reflex might be present. This implies that in case no nystagmus was found, the presence of a low-velocity nystagmus was not ruled out. Outcomes in this study were most likely not significantly influenced by this phenomenon, since in only 12% no nystagmus was found (Table 1) and the LED light of the Nystagmocatcher to illuminate the eye partially acts as a ‘penlight cover test’ (still partially disrupting visual fixation) [42]. Secondly, patients might not have perfectly performed the Dix-Hallpike maneuvers despite extensive guidance and help of a partner or friend (Table 3). It was hypothesized that this did not significantly influence the outcomes of this study, since the posterior canal was most likely sufficiently stimulated to detect BPPV. This was supported by two findings: (1) the presence of upbeat and torsional nystagmus during the head movement of the Dix-Hallpike maneuvers. This finding was congruent with stimulation of the posterior semicircular canal, and it was also present in video recordings of patients with negative Dix-Hallpike maneuvers; (2) the high rate of nystagmus found in this study. Thirdly, no lateral roll was included in the positional testing protocol. It was explicitly not included to decrease the burden of testing during an attack. Therefore, lateral canal BPPV might be missed in this study. Nevertheless, this study did not aim to precisely define the prevalence of specific disorders. The main objectives were to describe adherence, characteristics, and first clinical experiences regarding the applied protocol for capturing ictal nystagmus.

## Conclusion

Capturing ictal nystagmus can contribute to the diagnostic process in several ways, which motivates to rethink current clinical workflow in vestibular medicine. However, strict guidance is necessary and not all patients provide ictal recordings. In an outpatient setting, it would be advised to use ictal nystagmus recordings on indication, to complement the diagnostic process.

### Supplementary Information

Below is the link to the electronic supplementary material.

Supplementary file1 (DOCX 261917 KB)

## Data Availability

The participants of this study did not give written consent for their data to be shared publicly, so due to this supporting data is not available.
