# Supplementary material for: Capturing nystagmus during vertigo attacks using a smartphone: adherence, characteristics, pearls and pitfalls
Source: J Neurol. 2023 Aug 31;270(12):6044–56. doi: 10.1007/s00415-023-11965-y (PMC10632223; doi:10.1007/s00415-023-11965-y)
Supplement: Supplementary file 1 — Supplementary file1 (DOCX 261917 KB) [file 415_2023_11965_MOESM1_ESM.docx]

**Appendix/Supplementary materials**

To ensure patient privacy, the sound of the videos has been muted.

Case 1

Case 2

Case 3

Case 4

Case 5
